# Modeling Rice Metabolism: From Elucidating Environmental Effects on Cellular Phenotype to Guiding Crop Improvement

**DOI:** 10.3389/fpls.2016.01795

**Published:** 2016-11-29

**Authors:** Meiyappan Lakshmanan, C. Y. Maurice Cheung, Bijayalaxmi Mohanty, Dong-Yup Lee

**Affiliations:** ^1^Bioprocessing Technology Institute, Agency for Science, Technology and ResearchSingapore, Singapore; ^2^Department of Chemical and Biomolecular Engineering, National University of SingaporeSingapore, Singapore; ^3^Synthetic Biology for Clinical and Technological Innovation, Life Sciences Institute, National University of SingaporeSingapore, Singapore

**Keywords:** rice, metabolism, flux-balance analysis, genome-scale metabolic networks and models, systems biology, -omics data

## Abstract

Crop productivity is severely limited by various biotic and abiotic stresses. Thus, it is highly needed to understand the underlying mechanisms of environmental stress response and tolerance in plants, which could be addressed by systems biology approach. To this end, high-throughput omics profiling and *in silico* modeling can be considered to explore the environmental effects on phenotypic states and metabolic behaviors of rice crops at the systems level. Especially, the advent of constraint-based metabolic reconstruction and analysis paves a way to characterize the plant cellular physiology under various stresses by combining the mathematical network models with multi-omics data. Rice metabolic networks have been reconstructed since 2013 and currently six such networks are available, where five are at genome-scale. Since their publication, these models have been utilized to systematically elucidate the rice abiotic stress responses and identify agronomic traits for crop improvement. In this review, we summarize the current status of the existing rice metabolic networks and models with their applications. Furthermore, we also highlight future directions of rice modeling studies, particularly stressing how these models can be used to contextualize the aﬄuent multi-omics data that are readily available in the public domain. Overall, we envisage a number of studies in the future, exploiting the available metabolic models to enhance the yield and quality of rice and other food crops.

## Introduction

Rice (*Oryza sativa* L.) is a staple food consumed by more than half of the world’s population. Tremendous progress has been made in rice production during the past half century with a doubling of yield since the Green revolution in 1960s (Elert, 2014). This was achieved by the advancement and expansion of rice breeding system to develop high-yielding rice varieties, an increase in fertilizer use and a significant expansion of land area for rice cultivation. However, we are still facing the challenge of food shortage due to the sharp increase in global population as well as the negative effects of climate change on rice production (Wassmann et al., 2009; Dayton, 2014). To tackle this problem, a better understanding of rice physiology and metabolism is essential for improving rice production, for example by increasing photosynthetic efficiency and tolerance to abiotic and biotic stress conditions. Notably, rice has the unique ability to germinate and grow up to coleoptile under anoxic/prolonged soil flooding conditions. It is believed that this distinctive behavior could be due to the metabolic adaptation by active ethanolic fermentation although the underlying mechanism is still not completely well-understood. In addition, some varieties of rice have the adaptive mechanism to tolerate complete submergence with limited gas diffusion, low light and mechanical damage caused by flash flooding as well as deep water flooding (Jackson and Ram, 2003; Bailey-Serres et al., 2010). During such stress conditions, some rice cultivars have the ability to facilitate internal aeration by developing aerenchyma, leaf gas films and barrier to radial oxygen loss from the root depending on the type and depth of flooding (Nishiuchi et al., 2012). However, the distinctive mechanism that enables rice to tolerate such adverse conditions is yet to be revealed. To better understand these mechanisms, multiple approaches have been applied by rice scientists around the world starting from the elucidation of the whole rice genome sequence (International Rice Genome Sequencing Project, 2005) to high throughput -omics profiling together with advanced bioinformatics tools.

**FIGURE 1:**
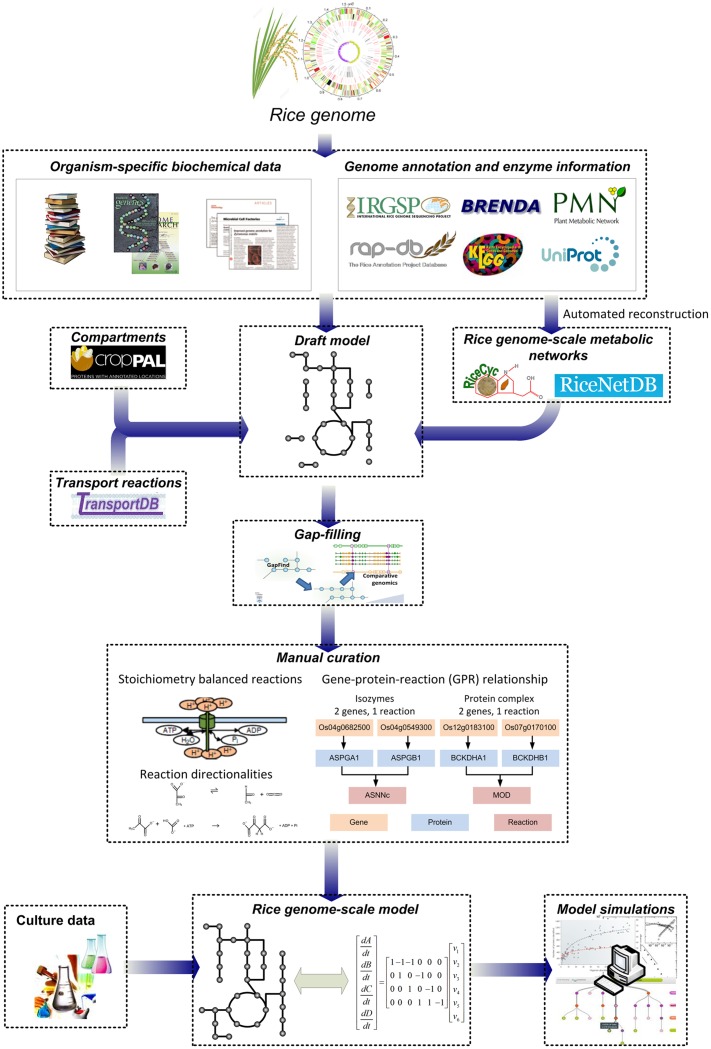
**Construction process of rice genome-scale metabolic networks and models.** Draft metabolic networks are first constructed based on information in the rice genome and various biochemical databases, followed by gap-filling and manual refinement to produce rice genome-scale metabolic networks (GSMNs). Further gap-filling, introduction of gene-protein-reaction associations and manual curation, compartmentation of the model, the addition of metabolite transporters, input and output reactions and model testing are required to build computation-ready rice genome-scale metabolic models (GEMs).

Concurrently, the recent advances in metabolic modeling approaches and the availability of genome information for numerous organisms have facilitated the reconstruction and analysis of genome-scale metabolic networks (GSMNs) in all three domains of life (Kim et al., 2012). Based on the genome of an organism of interest, a draft metabolic network can be developed by compiling the metabolic reactions catalyzed by enzymes encoded in the genome. Information on metabolites and reaction stoichiometry are typically obtained from multiple biochemical databases such as MetaCyc (Caspi et al., 2012) and Kyoto Encyclopedia of Genes and Genomes (KEGG) (Kanehisa et al., 2012). The draft network is then further gap-filled and manually refined to improve network connectivity by checking elemental balance and reaction directionality; gene-protein-reaction (GPR) associations are also included and exchange reactions are added for allowing nutrients uptake and secretion from the system. The reconstruction process of rice GSMN is summarized in **Figure 1**. Detailed procedures and challenges involved in the reconstruction process have been previously described and reviewed elsewhere (Thiele and Palsson, 2010).

Once the network is reconstructed, it can be readily converted into a mathematical model, which is represented in a matrix form where the rows and columns represent the metabolites and reactions, respectively. This “stoichiometric matrix” or simply “S matrix” is the centerpiece of constraint-based model reconstruction and analysis methodology where the stoichiometric coefficients constrain the flow of metabolites from various substrates to products in the metabolic network. In addition, measured extracellular uptake and secretion rates can be used as additional constraints. Together, these constraints confine a region within the multi-dimensional solution space of allowable reaction fluxes where the actual solution exists (Bordbar et al., 2014). Several techniques have been proposed to interrogate this solution space, among which constraints-based flux analysis, also known as flux-balance analysis (FBA), is the most commonly used approach (Orth et al., 2010). Basically, FBA optimizes a particular cellular objective function for determining the possible solution state within the metabolic network in terms of metabolic fluxes. Detailed information on metabolic modeling methodology is beyond the scope of this review and the comprehensive review could be found elsewhere (Lewis et al., 2012; Baghalian et al., 2014). Overall, the simplicity and extensibility of FBA and the availability of several conveniently accessible software tools to implement it (Lakshmanan et al., 2014a), genome-scale models have been extensively used for multiple applications including (i) prediction of metabolic phenotypes, (ii) investigation of metabolic network properties, (iii) contextualization of high throughput data, (iv) studying interspecies metabolic interactions, and (v) guidance of metabolic engineering (Oberhardt et al., 2009).

**FIGURE 2:**
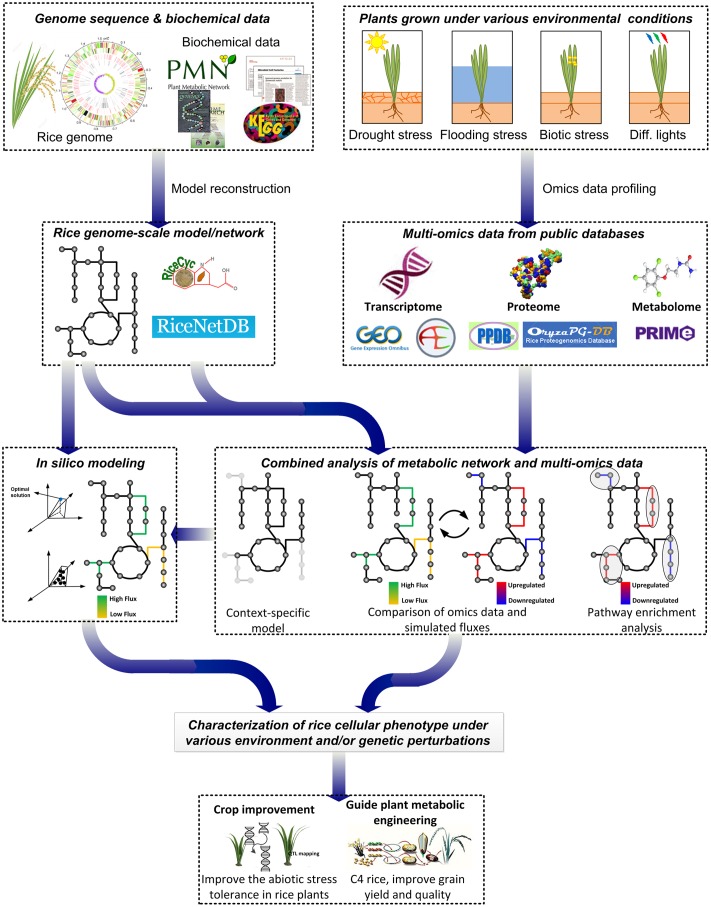
**Current and future applications of rice genome-scale metabolic networks and models.** Rice GSMNs/GEMs have been applied to contextualize -omics data to identify metabolic and transcriptional regulations. Metabolic fluxes were simulated using rice genome-scale metabolic models to investigate metabolic responses to different conditions. By combining modeling approaches of rice and other plant models, rice GSMNs/GEMs can be applied to guide engineering for improved rice production, to understand tolerance to abiotic stresses and to study metabolic interactions between rice and microbes.

In this review, we outline the current status and future prospective of the analyses and applications of rice metabolic networks and models (**Figure 2**). First, the existing rice metabolic networks and models are surveyed with their analyses and applications. Next, the potential and challenges in translating the modeling approaches applied and knowledge gained from other plant models to rice are discussed. Lastly, several future applications of rice genome-scale metabolic models are proposed for the improvement of rice production.

## Overview of Current Rice Metabolic Networks and Models

To date, two rice GSMNs (Dharmawardhana et al., 2013; Liu et al., 2013) and three rice genome-scale metabolic models (GEMs) (Poolman et al., 2013; Seaver et al., 2014; Lakshmanan et al., 2015) have been reconstructed based on multiple data sources. Here, we make a distinction between GSMNs and GEMs, similar to the definitions used in Lewis et al. (2012), where a GSMN comprises of all known metabolic reactions in an organism while a GEM is computable derivative of a metabolic network which can be analyzed using structural metabolic modeling and constraint-based modeling techniques such as FBA. The statistics of rice GSMN/GEMs are summarized in **Table 1**.

**Table 1:** Statistics of rice genome-scale metabolic networks and models.

	RiceCyc (Dharmawardhana et al., 2013)	RiceGEM (Liu et al., 2013)	Poolman et al. (2013)	PlantSEED (Seaver et al., 2014)	*i*OS2164 (Lakshmanan et al., 2015)
Number of genes	6643	4462	NA	1547	2164
Number of reactions	2190	3316	1735	1851	2441
Number of metabolites^∗^	1543	2986	1484	1841	1999
Number of compartments^#^	NA	10	3	9	7
Computation-ready	No	No	Yes	Yes	Yes
Number of allowed reactions	NA		853	1075	1723
Percentage of allowed reactions			49.2%	58.1%	70.6%
URL	http://pathway.gramene.org/ricecyc.html	http://bis.zju.edu.cn/ricenetdb/			

*^∗^Excluding external metabolites; ^#^Excluding ‘extracellular’ compartment.*

The two rice GSMNs, RiceCyc (Dharmawardhana et al., 2013) and RiceGEM (Liu et al., 2013), are accessible online as databases with information on genes, proteins and reactions. RiceCyc is a rice-specific metabolic pathway database constructed based on MetaCyc and plant metabolic network (PMN) (Chae et al., 2014). The initial stage of RiceCyc construction involved the functional annotation of rice genes using a number of data sources including InterPro (Mitchell et al., 2015), Gene Ontology (Ashburner et al., 2000), MetaCyc, Enzyme Commission (EC) numbers, KEGG and Gramene databases (Monaco et al., 2014). Notably, it contains information about genes, proteins and reactions across 316 metabolic pathways. However, it should be noted that RiceCyc does not provide any information on subcellular localization of metabolic reactions. In contrast, reactions in RiceGEM are assigned to nine subcellular compartments based on multiple protein localization prediction software (Liu et al., 2013). It was constructed by integrating reaction and metabolite information from various databases including RiceCyc, KEGG, Uniport (The UniProt Consortium, 2013) and Brenda (Chang et al., 2015). RiceGEM is available as a part of the RiceNetDB database^1^, which is a multi-level network database of rice created by integrating RiceGEM with gene regulatory networks and protein–protein interaction network.

The first computation-ready rice GEM was built from RiceCyc, with three subcellular compartments, cytosol, chloroplast, and mitochondrion (Poolman et al., 2013). Reactions in the cytosol were derived from RiceCyc, whereas reactions in the chloroplast and the mitochondrion and metabolite transport reactions were defined manually. The model was curated and checked for energy and redox conservation (no production of energy and redox equivalent from nothing) and stoichiometric consistency with respect to carbon, nitrogen, phosphorus, and sulfur (no production or consumption of C, N, P, and S from nothing). The next available rice GEM was constructed from the PlantSEED biochemistry database (Seaver et al., 2014), which integrates information from multiple sources including ModelSEED (Aziz et al., 2012), KEGG, PMN, MetaCyc-based databases and several published GEMs. PlantSEED supports the automated construction of computation-ready, gap-filled GEMs from plant genomes, currently with models of 10 plant species, including rice, available with gene-protein-reaction associations from the PlantSEED website^2^. Each PlantSEED model contains a comprehensive biomass reaction with 79 components and species-specific metabolic and transport reactions assigned to nine subcellular compartments mostly based on AraCyc (Mueller et al., 2003) and the Plant Proteomics Database (PPDB) (Sun et al., 2009). We tested the rice model from PlantSEED and found that the model has no stoichiometric inconsistency, but energy conservation is violated presumably due to the lack of manual curation in reaction reversibility. To the best of our knowledge, the most recent rice GEM is *i*OS2164 (Lakshmanan et al., 2015) reconstructed based on multiple databases including RiceCyc, PlantCyc, KEGG, and TransportDB (Ren et al., 2007). A distinct feature of this model is the detailed definition of all possible electron transport reactions in the mitochondrion, the plastid and the thylakoid including the light-driven photophosphorylation reactions in a wavelength specific manner, which allowed for the modeling of photosynthetic metabolism under different light sources. The model was manually curated for elemental balance, reaction reversibility and directions, assignment of reactions into seven subcellular compartments, gap-filling and gene-protein-reaction mappings. Energy is not conserved when considering the reaction stoichiometries, reversibility and directions of this model, but additional constraints were applied at the stage of model simulations to prevent spontaneous ATP generation (Lakshmanan et al., 2015). Here, it should be noted that while all GEMs represent rice metabolism, still the number of genes and reactions accounted vary drastically. Such inconsistencies arise due to the incompleteness in the data used for reconstructing the rice metabolic model. Therefore, in order to test the functional use of the models, we computed the number and the proportion of allowed reactions which can carry non-zero fluxes at steady-state and identified that *i*OS2164 has the highest proportion when compared to other three rice GEMs (**Table 1**), suggesting that this latest model has the broadest coverage of rice metabolism and supports the simulation of the highest number of metabolic functions in rice cells.

Apart from these rice GSMN/GEMs, a central regulatory/metabolic model of rice was also developed (Lakshmanan et al., 2013a), which covered all the central metabolic pathways. This model also included 52 direct and indirect regulatory interactions involving 12 regulatory proteins for differentiating the photosynthetic and non-photosynthetic cells based on Boolean logic formalism, controlling the activity of 40 metabolic reactions. Further, this model also has two manually curated biomass reactions, representing the rice seed/coleoptile and leaf based on literature information.

## Applications of Rice Metabolic Networks and Models

As GSMN/GEM represent systematic descriptions of the genotype–phenotype relationships, several *in silico* analyses were performed with/without multiple “-omics” data for investigating rice metabolism under both normal and stressed conditions. Here, we outline the applications of rice GSMN/GEMs including the contextualization of -omics data, followed by the characterization of rice metabolic responses to stress conditions and the investigation of the effect of light on rice metabolism.

### Characterizing Rice Metabolic Responses to Abiotic and Biotic Stresses

#### Flooding Stress

Predominantly, the developed rice metabolic models were used to unravel the metabolic adaptations of rice cells between normal and stressed conditions. For example, a core rice metabolic model successfully predicted and characterized the cellular metabolism of rice coleoptiles under air and anoxia (Lakshmanan et al., 2013a). The *in silico* simulation results showed the differential utilization of the glycolytic and ethanolic fermentation pathways based on oxygen availability and the use of sucrose synthase over invertase for efficient sucrose breakdown. Moreover, the study also highlighted the critical role of gamma-aminobutyric acid (GABA) in glycine synthesis via glutamate decarboxylase, succinic semialdehyde dehydrogenase and serine hydroxymethyltransferase, proposing the possible mechanism for observed anaerobic GABA accumulation (Shingaki-Wells et al., 2011). Later, the same model was used in conjunction with transcriptome data to explore the transcriptional regulation of cellular metabolism during anoxic adaptation (Lakshmanan et al., 2014b). Sucrose metabolism and fermentation were identified to be up-regulated, whereas oxidative phosphorylation, the tricarboxylic acid cycle and the pentose phosphate pathway were down-regulated at both the transcriptional and the metabolic flux levels, leading to the hypothesis that these pathways are transcriptionally regulated in response to anoxic conditions. The *cis*-regulatory content analyses of these transcriptionally controlled enzymes further revealed the combined regulatory role of four transcription factors, namely, MYB, bZIP, ERF, and ZnF.

#### Drought Stress

The core rice metabolic model was also applied to investigate the drought stress responses by simulating the cellular metabolism at various levels of carboxylation/oxygenation ratios of Rubisco (Lakshmanan et al., 2013a). The model simulations highlighted the important roles of the plastidial malate-glutamate and malate-oxoglutarate transporters in recycling ammonia from the photorespiratory pathway and the mitochondrial malate-oxaloacetate transporter in shuttling excess reductant out of the mitochondrion. Additionally, *in silico* analysis showed that oxidative phosphorylation was crucial during drought stress as ATP generated in mitochondrion was exported to the plastid to power the Calvin-Benson cycle, the glutamine synthetase-glutamate oxoglutarate aminotransferase cycle and the plastidic part of the photorespiratory pathway. In addition, Lakshmanan et al. (2013b) identified a number of essential genes/reactions of rice under photorespiratory condition, which were in good agreement with experimental results, as well as more than 200 synthetic lethal gene pairs (pairs of non-essential genes whose simultaneous deletion become lethal) in rice central metabolism. Similar to this analysis, another study analyzed the essential reactions and the effect of photorespiration on chlorophyll synthesis using the previously published GEM (Chatterjee and Kundu, 2015). More recently, Mohanty et al. (2016) integrated the gene expression data onto RiceCyc GSMN, thereby elucidating the drought-induced changes in cellular metabolic pathways due to transcriptional regulation. Further, based on such differentially regulated genes, they also highlighted several critical transcription factors which could be targeted for genetic selection in drought tolerance breeding. Noticeably, most of the model identified targets were previously linked to drought tolerant phenotypic variations.

#### Biotic Stress

The metabolic changes of rice induced by pathogens were investigated by mapping gene expression data for pathogen induction in rice to the RiceCyc metabolic network (Dharmawardhana et al., 2013). Several genes from the biosynthetic pathway of tryptophan, a metabolic precursor for plant defense-related secondary metabolites, showed widespread induction by a range of different pathogens including bacteria, fungi and an angiosperm parasitic weed. The analysis was extended to serotonin and auxin biosynthesis and it was found that the rate-limiting step of serotonin biosynthesis was strongly induced by a broad spectrum of pathogens but not for the other enzymes in serotonin biosynthesis pathway, whereas auxin biosynthesis genes were not broadly induced under biotic stresses.

### Investigating the Effect of Lights on Rice Metabolism

#### Diel Cycle

The global transcriptional regulation of metabolic and transport genes over a day–night cycle was investigated through the contextualization of public transcriptome datasets on diel regulation of rice plants onto the RiceCyc metabolic network (Dharmawardhana et al., 2013). The analysis identified 2,225 metabolic and transport genes whose transcriptions were under diel regulation, among which the biosynthesis of amino acids, nucleotides and nucleosides, carbohydrates and cell structure components was highly active just before dusk; the fatty acid and lipid biosynthetic processes showed activation during dawn. Regarding secondary metabolites, lignin metabolism, mevalonate and momilactone pathways as well as the biosynthesis of plant growth hormones gibberellin and auxin showed activation during dawn.

#### Light Intensity

Poolman et al. (2013) developed a rice GEM to simulate the responses of rice metabolism to varying light intensities. The shuttling of redox metabolites between the chloroplast, cytosol, and mitochondrion was shown to play a critical role in maintaining cellular homeostasis, particularly at low light levels. The analysis also indicated the usefulness of photorespiration as a drain for excess energy at high light intensities and the importance of mitochondrial metabolism in supporting photosynthesis where the mitochondrial ATP generation was modulated according to the light intensity. A follow-up analysis modeled the metabolic trade-offs between growth and photosynthate export in rice leaf, which demonstrated the robustness of flux patterns in central carbon metabolism with little changes in fluxes between an expanding leaf and a mature leaf (Poolman et al., 2014).

#### Light Quality

The latest rice GEM, *i*OS2164, was utilized in conjunction with transcriptomics and metabolomics data to characterize the cellular metabolism of rice under four different light spectrums, red, green, blue and white, and in the dark (Lakshmanan et al., 2015). It was shown that several metabolic pathways in rice responded differentially to certain light colors where photosynthesis and secondary metabolism were up-regulated in blue light, whereas carbohydrates degradation was pronounced in dark. Through the mapping of transcriptome data onto *i*OS2164, followed by a topological analysis of the metabolic model, the key biomarkers for light-mediated transcriptional regulation were identified, including several phytohormones such as ethylene, gibberellin, and jasmonate (Lakshmanan et al., 2015). Enzymes involving the identified biomarker metabolites were extracted from the model and the promoter regions of the genes of these enzymes were subsequently analyzed, which pointed to several light-specific putative *cis*-regulatory elements and their cognate transcription factors. Moreover, the authors have shown that the proposed model-driven framework could correctly identify the possible light specificity of several transcription factors. By combining metabolome and transcriptome data with *i*OS2164, key metabolic and regulatory signatures between rice leaves grown in red light and blue light were further elucidated.

#### Role of Alternate Electron Flow Pathways

Noticeably, the *i*OS2164 GEM was also utilized to evaluate the role of different alternative electron flow (AEF) pathways during photosynthesis and cell growth and identified that all three electron flow pathways are possibly operational at all times (Lakshmanan et al., 2015). Particularly, this study highlighted that the role of AEF pathways, especially cyclic electron flow become significant under high light and low carbon conditions for dissipating the excess redox power that are generated by the photosystems.

## Translating Modeling Approaches from Other Plant Models to Rice

Besides studies on rice metabolic models, there have been numerous publications on large-scale metabolic modeling of *Arabidopsis* and other plants in the past few years. Thus, we review the studies on plant metabolic modeling which are relevant and applicable to rice metabolic models.

### Modeling Seed Metabolism

The main goal in studying crop plant metabolism is to improve the yield and the quality of grains (seeds). The most direct approach is to understand the metabolism during seed development. Thus, several studies using constraint-based modeling were carried out to model metabolism in barley seeds (Grafahrend-Belau et al., 2009) and oilseed rape embryos (Hay and Schwender, 2011a,b; Borisjuk et al., 2013). The growth and metabolic fluxes of developing endosperm of barley in response to oxygen availability from completely anaerobic to aerobic were simulated and *in silico* enzyme knockout analysis was performed which demonstrated the flexibility of plant metabolism to compensate for enzyme perturbations (Grafahrend-Belau et al., 2009). Recently, the same model was also used to analyze the metabolic architecture of barley seeds and identified that these seeds possess a remarkable high carbon conversion efficiency of more than 95% (Rolletschek et al., 2015). Using flux variability analysis, the metabolic behavior of developing oilseed rape under four nutritional conditions was investigated, which indicated the metabolic redundancy and flexibility of the system (Hay and Schwender, 2011b). Fifty-seven reactions were identified with function in metabolic adjustments to different nutritional conditions based on the results from flux variability analysis. There was a high correlation between the model flux prediction and experimental flux map (Hay et al., 2014), though the model failed to predict fluxes through the oxidative pentose phosphate pathway and the use of alternative carbon and nitrogen sources (Hay and Schwender, 2011a). The modeling results revealed alternative pathways for the production of pyruvate and NADPH for fatty-acid synthesis and an unexpected role of glycine decarboxylase in ammonia assimilation. With the aim of manipulating seed composition, the carbon allocation into seed storage compounds was analyzed by simulating the tradeoff between oil and protein accumulation in developing oilseed rape embryos to identify reactions responsive for oil or protein production (Schwender and Hay, 2012). Glycolytic reactions were found to be the most oil responsive whereas reactions for mitochondrial ATP synthesis were the most protein responsive. The same model was further applied to investigate the seed metabolic architecture and identified that the fluxes are locally regulated to efficiently use the available light and space (Borisjuk et al., 2013). Clearly, similar modeling approaches could also be applied to rice metabolic models to investigate the flexibility and behavior of the metabolic system in developing rice grain and to identify metabolic changes required for the manipulation of the composition of rice grains.

### Metabolic Responses to Environmental Changes

The existing rice GEMs have been used to investigate the effects of the changes in light quantity or quality on metabolic fluxes in leaves (Poolman et al., 2013, 2014; Lakshmanan et al., 2015). A central regulatory/metabolic model of rice was applied to simulate the metabolic responses to anoxia and photorespiratory conditions (Lakshmanan et al., 2013a,b). Besides such analyses on rice models, metabolic responses to various environmental conditions were simulated with other plant models, in particular the metabolic models of *Arabidopsis*. The metabolic fluxes in *Arabidopsis* heterotrophic cell culture under increased temperature and hyperosmotic conditions were accurately predicted using *Arabidopsis* GEMs (Williams et al., 2010). Using a diel GEM of *Arabidopsis* (Cheung et al., 2014), the effects of varying light intensity or nitrogen uptake on the metabolism of mature leaves over a day–night cycle were investigated. By integrating GEM and transcript profiling data, Töpfer et al. (2013) characterized the metabolic response under eight light and/or temperature conditions and identified relevant metabolic pathways involved in light and temperature acclimation in *Arabidopsis*. Using a maize leaf GEM, the metabolic impact of nitrogen availability was modeled with condition-specific biomass compositions and regulatory constraints based on transcriptomic and proteomic data (Simons et al., 2014). These studies successfully demonstrated the applicability of GEMs in simulating metabolism under different environmental conditions. Thus, further analyses on metabolic responses to simultaneous changes in multiple environmental parameters using rice GEMs could provide valuable insights into the key metabolic processes important for adaptation to specific environmental field conditions for optimal growth and crop yield.

### Metabolic Models as Scaffolds for Integrating Multiple -omics Data

One of the key applications of GSMN/GEMs is to discover new knowledge through its integration with multiple -omics data. Demonstrated by a number of studies on PMNs and models, there are several approaches in integrating -omics data with GSMN/GEMs: (1) it can be contextualized in metabolic networks and models, for example transcriptomic data was mapped to rice networks and models to determine the regions of the metabolic system which are significantly perturbed (Dharmawardhana et al., 2013) or under transcriptional regulation (Lakshmanan et al., 2015); (2) transcriptomic and/or proteomic data can be converted into model constraints for various simulations of reaction fluxes (Töpfer et al., 2013, 2014; Schwender et al., 2014; Simons et al., 2014; Lakshmanan et al., 2015) and (3) changes in predicted reaction fluxes and metabolite turnover rates between any two conditions can be compared with that of gene expression and metabolome data (Töpfer et al., 2013, 2014; Lakshmanan et al., 2015). Among these different methods, the use of gene expression/proteome data as additional reaction constraints is promising because plant metabolic models are intrinsically large, with multiple parallel reactions and pathways, which often lead to variability in model flux predictions. In fact, more than 10 *in silico* algorithms were developed for integrating transcriptomic data with GEMs (Blazier and Papin, 2012; Hyduke et al., 2013; Machado and Herrgård, 2014). Using such approaches, GEMs of *Arabidopsis* (Töpfer et al., 2013, 2014), rice (Lakshmanan et al., 2015) and maize (Simons et al., 2014) have effectively utilized transcriptomic and/or proteomic data as surrogates to regulatory constraints in constraints-based flux analysis for simulating the functionally active metabolism under different environmental conditions. Besides using -omics data for model analyses, transcriptomic and proteomic data can be utilized in the construction process of GEMs for model compartmentation (Simons et al., 2014) and generation of tissue-specific models (Mintz-Oron et al., 2012).

### Beyond a Generic Single-Cell GEM

Thus far, most available GEMs of C_3_ plants are generic single-cell models for simulating the metabolism of a single cell or tissue. The published rice GEMs were used to model growing photosynthetic leaves based on tissue-specific biomass compositions (Poolman et al., 2013; Lakshmanan et al., 2015). Amongst the numerous published *Arabidopsis* GEMs, only one study constructed multiple tissue-specific models, representing 10 *Arabidopsis* tissues based on proteomics data (Mintz-Oron et al., 2012). In a core metabolic model of rice, tissue-specific biomass compositions as well as tissue-specific constraints on enzymatic reaction fluxes based on transcriptomic data were applied to model the metabolism in rice coleoptile under anoxia and in leaf under photorespiratory condition (Lakshmanan et al., 2013a,b, 2014b). It should be noted that the application of tissue-specific constraints on enzymatic reaction fluxes on a generic metabolic model is functionally similar to the use of tissue-specific models. Further, many of the *Arabidopsis* GEMs have been applied successfully to model multiple cell types or tissues under different conditions with relevant tissue-specific input and/or output constraints and an appropriate objective function. Therefore, it may be recommended that in the absence of detailed tissue specific gene or protein expression data, just the physiological information about specific tissues/conditions can be formulated as appropriate constraints and/or objective functions to predict the intracellular metabolism reasonably well.

Metabolic interactions between two cell types in C_4_ leaves, bundle sheath cell and mesophyll cell, have been modeled using GEMs of C_4_ plants (de Oliveira Dal’Molin et al., 2010; Simons et al., 2014). By allowing metabolite exchanges between the two cell types, the C_4_ models successfully predicted the operation of these pathways of the three C_4_ subtypes. Analogous to modeling interactions between two cell types, metabolic interactions between light and dark metabolism in a C_3_ leaf was modeled using a diel modeling framework which divides leaf metabolism over a day–night cycle into two steady-states, a light state and a dark state (Cheung et al., 2014). The application of the diel modeling framework to rice GEMs will allow for more accurate simulations of photosynthetic metabolism of rice over a day–night cycle.

Genome-scale metabolic models have been successful in simulating plant metabolism at a single cell or tissue level by applying cell- or tissue-specific input and output constraints and/or objective functions which define the metabolic function(s) of the cell or tissue. However, a plant cell or tissue does not operate in isolation but is dependent on other cells, tissues and organs. Hence, there is a need to extend the existing genome-scale metabolic modeling framework to a whole-plant scale. To this front, a multi-scale metabolic modeling approach integrating a dynamic whole-plant functional model with a multi-organ flux-balance metabolic model was developed to study the source-sink interaction during seed development in barley plants (Grafahrend-Belau et al., 2013). Using dynamic FBA, the metabolic dynamics of barley at a whole-plant level was modeled which revealed a sink-to-source shift of barley stem due to a senescence-related decrease in leaf source capacity. This whole-plant modeling approach could be applied to GEMs of rice and other plants to study the metabolic dynamics and source-sink interactions of various plant tissues over the course of plant development from seed germination to seed development.

## Challenges in Constraint-Based Modeling of Plant Metabolism

Influenced by the history of FBA and genome-scale metabolic modeling from bacterial systems, most plant flux-balance modeling studies simulated the metabolism of cell or tissue growth by setting a constraint on the synthesis of biomass components or an objective function of maximizing biomass synthesis. While growth as a metabolic function is applicable for plant cell or tissue cultures, developing seeds and young leaves, there are many plant tissues and organs, such as mature and senescing leaves, maturating fruits, etc., which have distinct metabolic behavior and phenotypes other than growth. In order to apply genome-scale metabolic modeling to more plant tissues, the definition of tissue-specific metabolic function(s) must be extended beyond cell or tissue growth. One example is the use of an *Arabidopsis* GEM to simulate the metabolism of mature (non-growing) C_3_ and CAM leaves by defining the metabolic function of mature leaves with model constraints of sucrose and amino-acids export into the phloem (Cheung et al., 2014). Fruit maturation is another example where growth is not applicable, demonstrated by a recent FBA study on modeling the metabolic shifts of nine sequential stages of tomato fruit development using the objective function of flux minimization, constrained by measured concentrations of biomass components and accumulated metabolites in the pericarp at each stage (Colombié et al., 2015).

One of the major challenges in GEMs is the incorporation of regulatory aspects into the modeling framework. To do so, Boolean logic formalism was incorporated in one of the studies to control the reaction activity of rice central model, accounting the regulation of seed germination under aerobic and anaerobic conditions (Lakshmanan et al., 2013b). Here, it should be noted that although such an approach was shown to be successfully applied for modeling different tissue/conditions, it still has certain limitations: it just provides two possible states for target metabolic reactions, i.e., ON or OFF. However, in reality, biological systems exhibit a wide range of responses in transcriptional regulation, from binary to even continuous. To address such issues, alternative methods such as probabilistic regulation of metabolism (PROM; Chandrasekaran and Price, 2010) have been devised for the continuous integration of cellular regulatory network onto the genome-scale model using abundant transcriptomic data and regulatory interaction information, resulting in better combined regulatory-metabolic models. As an alternative, gene expression data can also be used as surrogate for cellular regulation to consider only the active metabolic pathways. Several methods such as Gene Inactivity Moderated by Metabolism and Expression (GIMME; Becker and Palsson, 2008), Integrative Metabolic Analysis Tool (iMAT; Shlomi et al., 2008), and E-Flux (Colijn et al., 2009) have been proposed to achieve this feat. While numerous other methods are still being developed in this regard for the appropriate integration of gene expression data onto GEM, such approach has already been successfully adopted by several plant modeling studies including rice (Mintz-Oron et al., 2012; Töpfer et al., 2013; Lakshmanan et al., 2015).

Another problem that stemmed from the mentality of simulating growth is the bias in manual curation of GEMs for reactions involved in synthesis of core biomass components (i.e., carbohydrates, proteins, nucleic acids, lipids). Most plant metabolic models were tested for biosynthesis of biomass components but relatively little effort was made in their catabolism, which is important for modeling senescing plant tissues. While carbohydrate and lipid degradation were included in most plant metabolic models, this is generally not the case for catabolism of other biomass components such as amino-acids, nucleic acids and chlorophyll. With respect to secondary metabolism, although the biosynthesis of a limited set of secondary metabolites was curated for a number of plant GEMs, the vast majority of complex plant secondary metabolites could not be produced from the models. Furthermore, the biosynthesis of most secondary metabolites was neglected in model simulations, primarily due to the lack of experimental data on the production of secondary metabolites. The exclusion of secondary metabolites from the model constraints or biomass equations could have an impact on the results from various analyses such as gene essentiality and energetic costs calculations.

Plant metabolic models were applied to simulate metabolism under multiple abiotic conditions. While a number of abiotic factors directly relate to metabolism and can be captured directly by constraints in flux-balanced models, such as anoxia and light intensity, some require indirect proxies to translate the abiotic condition into model constraints. For example, Rubisco carboxylation to oxygenation ratio was used as a proxy for stomatal closure to simulate drought stress (Lakshmanan et al., 2013a). In addition many conditions have no simple relation to the metabolic system, e.g., heat and cold stresses, hyperosmotic conditions and salt stress, therefore model simulations of these conditions require additional information such as transcriptome data (Töpfer et al., 2013) or extensive experimental measurements for model constraints (Williams et al., 2010). The continuous progress in tackling these challenges in plant metabolic modeling will enable us to extend the scope of future applications of GEMs of rice and other plants.

## Future Applications of Rice Gsmns/Gems

The primary aim of rice genome-scale metabolic modeling is to enhance our understanding of rice metabolism to guide its engineering and manipulation for improved quality and quantity of rice grain production. One active research area is the engineering of rice by incorporating C_4_ photosynthesis, at both biochemical and anatomical levels, into rice (Hibberd et al., 2008; Zhu et al., 2010; von Caemmerer et al., 2012). *In silico* engineering of C_4_ rice can be performed using rice GEMs to simulate the optimal metabolic distribution for efficient photosynthesis in C_4_ rice. This will allow the identification of metabolic manipulations required for optimizing C_4_ photosynthesis in rice, which include not only enzymes in the C_4_ pathway but also other related processes such as the shuttling and balancing of ATP and reductant to meet altered metabolic demand and the localization of carbon storage to support dark metabolism. Besides C_4_, there have been suggestions in engineering CAM photosynthesis into C_3_ crop plants to improve photosynthetic and water use efficiency for sustaining and improving crop productivity in a warmer and drier world (Borland et al., 2014). The effect of engineering CAM photosynthesis into rice can be modeled by applying a diel modeling framework (Cheung et al., 2014) on rice GEMs, which could guide the engineering and optimization of CAM photosynthesis in rice.

In addition to the efforts in increasing the productivity of rice, work has also been done on improving the nutritional quality of rice grains (Bhullar and Gruissem, 2013). The most widely known example is the golden rice, which is engineered to produce β-carotene (pro-vitamin A) in the endosperm to combat vitamin A deficiency (Beyer et al., 2002; Paine et al., 2005). Other examples include engineering higher levels of folate, iron and essential amino-acids, etc. in rice grains (Bhullar and Gruissem, 2013). The biosynthesis of metabolic products with high nutritional values in rice can be investigated using rice GEMs, which may give hints to possible strategies for producing rice grains with increased nutritional value.

Up until now, plant flux-balance models have mostly been applied to investigate metabolism under different abiotic conditions, with relatively little work done on modeling biotic conditions. A well-known example of plant–microbe interaction is legume–rhizobia symbiosis. Using GEMs of a legume (*Medicago truncatula*) and a rhizobium (*Sinorhizobium meliloti*), the metabolic interactions underlying the symbiotic relationship between the two organisms were modeled, suggesting that oxygen availability limits symbiotic nitrogen fixation and influences the form of nitrogen supplied to the plant (Pfau, 2013). Regarding interactions between rice and microbes, rice pathogens can cause up to 50% yield loss, resulting in large economic losses (Miah et al., 2013). To understand the manipulation of rice metabolism by pathogens during pathogenesis, metabolic models of rice pathogens such as *Xanthomonas oryzae* pv. oryzae and *Magnaporthe oryzae* can be developed and integrated with rice GEMs to investigate rice–pathogen metabolic interactions, similar to a study on modeling human–pathogen metabolic interactions (Bordbar et al., 2010). Analysis of an integrated rice–pathogen model could potentially identify strategies to counteract the metabolic manipulation by the pathogens for engineering disease-resistant rice strains.

While GEM on its own is already a powerful tool for investigating the behavior of metabolic systems, a lot more can be done by making use of the gene-protein-reaction associations in the models, illustrated by a number of plant modeling studies ( Töpfer et al., 2013, 2014; Schwender et al., 2014; Simons et al., 2014; Lakshmanan et al., 2015). As different types of -omics data are becoming more readily available, GSMN/GEMs will prove to be important tools for analyzing and contextualizing multiple -omics data. Further work on integrating multiple -omics data with rice GSMN/GEMs will lead to better understanding of the regulation of rice metabolism, which is crucial for improving crop production.

## Summary

In summary, the development and application of rice GSMN/GEMs in recent years have increased our understanding of the metabolic and transcriptional regulations and the metabolic responses of rice to multiple environmental conditions and stresses through the integration of -omics data and/or constraint-based modeling. As the field of plant metabolic modeling progresses, modeling approaches developed for *Arabidopsis* and other plant models will undoubtedly be applied to rice models to better understand the metabolic systems of this important crop plant. While there are several limitations and challenges in applying constraint-based techniques in modeling plant metabolic systems, progress are being made in overcoming these limitations and challenges, which will extend the scope of future applications of GEMs of rice and other plants. With the ever increasing availability of multiple -omics data and experimental measurements, we envision that rice GSMN/GEMs will play a key role in contextualizing and analyzing these complex data to give novel insights into rice metabolism, which will lead to improvements in the yield and quality of rice production.

## Author Contributions

ML, CC, and D-YL have contributed in the design and write up of the manuscript. ML, BM, and D-YL have edited, reviewed, and approved the final version of the manuscript.

## Conflict of Interest Statement

The authors declare that the research was conducted in the absence of any commercial or financial relationships that could be construed as a potential conflict of interest.

## Footnotes

1 http://bis.zju.edu.cn/ricenetdb/

2 http://plantseed.theseed.org/
